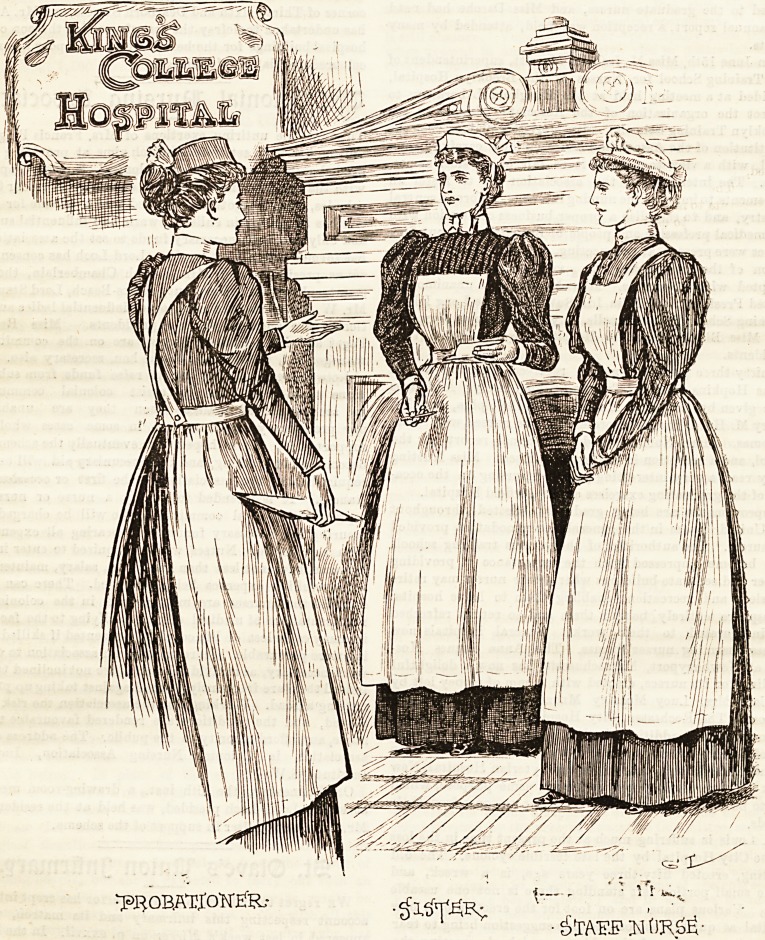# "The Hospital" Nursing Mirror

**Published:** 1896-07-18

**Authors:** 


"The Hospital" Nursing Mirror.

Being the Extra Nursing Supplement of "The Hospital" Newspaper.

[Contributions for this Supplement should be addressed to the Editor, The Hospital, 428, Strand, London, W.C., and should have the word "Nursing" plainly written in left-hand top corner of the envelope.]

### News from the Nursing World.

### PRINCESS VICTORIA OF WALES.

The arrival of Prince Charles very naturally accounted for the absence of Princess Maud from her sister's side at the opening of the new Charing Cross Hospital Convalescent Home last Saturday. We are so accustomed to the presence of both the princesses at functions in which their parents take part, that her absence on the above occasion reminded us that Princess Victoria will, in the future, be without the assistance of her sister on public occasions. Princess Victoria has always borne a singularly happy part in the home life of the Royal household. She has earned for herself so warm a place in the hearts not only of her relatives, but of every member of the household, that she is in thought known to everybody as the angel of the family. Princess Victoria's devotion to her mother, and the deep affection which unites parent and child are incidents too precious to dwell upon in this place. It must suffice to say that whenever there has been sorrow to bear, suffering to alleviate, or painful tasks to undertake, then Princess Victoria has always been to the fore and has ever exhibited the tenderest tact and the most self-denying devotion. Beloved by everybody, knowing from bitter experience what suffering is, she has cultivated a cheerful spirit, and is in fact the sun which warms, strengthens, and unites the hearts of all who come within the sphere of her influence.

### THE ROYAL HOSPITAL, RICHMOND.

The new Princess May Ward of the Richmond Royal Hospital was formally opened on Wednesday in last week by the Duchess of York. The Duke and Duchess of York, with whom were the Duke and Duchess of Teck, were received by the Duke of Cambridge as president of the hospital. In the marquee, to which the Royal party were escorted, Viscount Middleton made a short speech of welcome, and bouquets were presented to the Duchess of York and the Duchess of Teck, after which the chairman of the hospital (Colonel R. W. Sparks) read an address. The Duke of York expressed the pleasure felt by himself and the Duchess in opening the new children's ward, which they trusted would be very helpful to the district. They cordially wished the hospital a long and prosperous career. The Duchess especially had lived so long in Richmond that she naturally took the deepest interest in its charitable institutions. This additional ward and an isolation ward have cost £6,250, the whole of that sum having been contributed. Purses containing some £300 were received by the Duchess on the opening day, and with a few other donations it has been possible to proclaim the highly satisfactory news that the new ward was opened free of debt, a result upon which all connected with the hospital and its management must be heartily congratulated.

### NEW NURSES' HOME AT PAISLEY.

Last week the new home for the nursing staff of the Paisley Infirmary, built at the expense of Mr. Peter Coats, of the Paisley Thread Works, was formally handed over to the directors of the infirmary by the donor. The new building is three-storeyed, and provides accommodation for twenty-eight nurses, the bed-rooms, sitting-rooms, and library being charmingly furnished. Mr. Coats said he had been prompted to provide the home because he saw the directors were unable, on financial grounds, to give the best accommodation to their nurses when building their new infirmary, and he was deeply impressed with the importance of such a home to those women who devoted their lives to the care of the sick. Mr. George Coats, chairman of the directors, who accepted the gift, remarked that the benefit the nurses would derive from their charming sitting-rooms and airy bed-rooms would be reflected on the patients, and the cheerful healthiness of the home would result in the increased efficiency of the whole staff.

### CONVALESCENT HOMES.

It is a pity that convalescent homes so often fail to accomplish much of the good they might do, because the unfortunate inmates, often only just recovering from more or less severe illness, are expected to do an amount of domestic work which entirely prevents the longed for "change of air" having its due effect. If the patients are expected to do all the "washing up," make their beds, sweep and dust, and, in fact, do all the cleaning of the establishment, they might as well have stayed at home. When it is considered, too, that in most convalescent homes a considerable payment is made either in the form of a letter of admission or weekly payment, it does not seem a fair bargain at all. That the people who make the largest use of these institutions are of the class which is accustomed to work of the kind is no reason for this too prevalent custom. All the more do they need the rest of doing nothing, like the lady in the epitaph who "always was tired," for they too "live in a place where too much is required."

### NURSES FOR COLLIERY CENTRES.

Dr. Haldane, in his recent report on mining explosions, in which he expresses his belief that the large proportion of deaths in explosions result not from the immediate effect, but from the slowly acting poison of carbon monoxide in the after-damp, points out the need that exists for prompt nursing aid for the victims of these terrible accidents. The mining villages in South Wales and the Black Country are frequently far away from such skilled service; hospital accommodation in such districts is limited, if not altogether absent, and nurses are not to be had. Something might surely be done to ensure a speedy ambulance service, and an adequate supply of the necessaries for dealing with cases of monoxide poisoning and severe burns and scalds in these parts of the country, and now that the want of such a service has been brought before public attention, doubtless steps will be taken for its supply.

### SUMMER EXPEDITIONS.

For several years past the nurses of University College Hospital have had a pleasant day's expedition organised for their benefit during the summer, thanks to the kindly energy of the secretary, Mr. Newton Nixon. The plan originated in a small way by a desire on Mr. Nixon's part to provide some little pleasure for the nurses, and at first this took the form of a concert. Next year the entertainment took a wider scope, and has finally grown to the dimensions of a day on the river for the entire nursing staff. The cost is defrayed by subscriptions, willingly forthcoming from friends of the hospital, the council of University College, and some of the tradespeople, expenses working out at the small sum of about 10s. a head. Brakes are hired to convey the nurses to Richmond, where a substantial luncheon is provided, and an electric launch chartered for the day's amusement. The night nurses join the party later, driving down in time for tea, and a trip in the launch, all returning together after supper. In order to enable the whole staff to participate in the fun, the All Saints' Sisterhood, by whom the University College Hospital is nursed, call in their private nurses to carry on the work of the wards, which is lightened for that day by the visiting physicians and surgeons forbearing to make their usual rounds, or at least doing so with less than customary ceremony. The funds for this pleasant expedition are, with a little personal energy and trouble, easily collected. This year, for various reasons, the University treat is not coming off, but to show the readiness with which the request for help is met, several subscribers have proffered their usual contributions. Other hospitals, both in London and in the provinces, might with advantage follow so good an example, and originate, according to their own ideas, some similar yearly outing for their nursing staff.

### "GOD HELP THE POOR PATIENTS."

"Then I may rely upon your daughters each giving us one day a week?" remarked Lord X, with a parting smile. As soon as the door had closed behind him the hostess turned to her husband; "I really call this quite providential," she said. "Well, I own I don't see what you are driving at," he retorted, with a bluntness which distressed her. "I have always wanted the girls to go in for philanthropy," she continued blandly, "it is so fashionable nowadays, but I feared it would cost too much time and money. Dear Lord X's plan has solved the difficulty, for he is getting together a corps of really nice people who will each give one day a week to a hospital. They are to take the place of the common regular nurses, and give them all a day's holiday in turn," then the lady lowered her voice, "and, quite between ourselves, my dear, it will be such an easy way for our girls to acquire a little professional knowledge without our having to pay fees to anyone for teaching them!" "But," urged the startled father, "will the doctors countenance such a plan, and how will the patients like it?" The hospital matron and her staff echoed his words when they heard of the new scheme, and the medical officer added, "God help the poor patients!"

### A DAY AT LLANDUDNO FOR LIVERPOOL NURSES.

Mr. George Grierson has written to the committees of the Liverpool city hospitals, asking permission to invite the nurses from each institution to Llandudno for a day's change and fresh air. Resolutions were unanimously passed accepting Mr. Grierson's kind offer with thanks. It is delightful to think of the pleasure which will be given to many hardworking women by this kindly thought.

### CHICAGO MIDWIVES AND THE HEALTH DEPARTMENT.

The Department of Health of the City of Chicago lately drew attention to the fact that it was becoming increasingly common amongst the midwives practising in Chicago to assume the conduct of serious cases, with a consequent rise in the mortality of both mothers and infants. Regulations have been promptly adopted to restrict midwives to attendance on cases of normal labour only; and as regards after attendance they are required in the future to be under medical control and supervision, which may be prescribed by the health authorities of the district in which they live or practice.

### MELBOURNE DISTRICT NURSING SOCIETY.

Lady Brassey takes a keen interest in charitable and philanthropic institutions. She visited the new home of the Melbourne District Nursing Society the other day and expressed herself much pleased with all its arrangements. Lady Clarke, the vice-president, and Miss Beck, the hon. secretary, presented the nurses to Lady Brassey; afterwards tea was served and a musical entertainment followed. The Home was very prettily decorated for the occasion, and a number of visitors accepted the invitations of the committee to be present.

### KENDAL NURSING ASSOCIATION.

The annual meeting of this district nursing association was held the first week in June, and the annual report showed that Nurse Oddy's labours among the poor of the district had been much appreciated. She had made 2,138 visits during the year, the demands upon her time and skill having been very heavy during the winter, when there had been much sickness. The people of Kendal and the neighbourhood do not seem to contribute as liberally as they ought to do towards the association, and it was suggested that unless more money could be raised, matters would end either by Nurse Oddy's salary of £70 being reduced, or by engaging "someone cheaper." Either course would be equally undesirable, rather should the present nurse look to some increase of pay for length of good service. Surely the needful funds to carry on a good work will not be asked for in vain.

### SHORT ITEMS.

At a meeting of the Notts Board of Guardians the other day it was decided to raise the subscription to the Northern Workhouse Nursing Association from one guinea to three guineas annually. The chairman at the same time referred to the advantage of obtaining their nurses through this source, experienced in workhouse nursing. The six nurses lately supplied by the association were doing good and useful work.—A district nursing association has been formed in Dunoon, largely through the efforts of ex-Provost Mackay, who is much interested in the movement.

### Hygiene: For Nurses.

By John Glaister, M.D., F.F.P.S.G., D.P.H.Camb., Professor of Forensic Medicine and Public Health, St. Mungo's College, Glasgow, &c.

### XV.—WATER SUPPLY (*Continued*).—CHARACTERISTICS OF POTABLE WATERS.—EFFECTS OF IMPURE WATER ON HEALTH.

A pure and wholesome water ought to be (1) colourless, (2) tasteless, (3) odourless, (4) bright, sparkling, and clear, (5) to have little mineral substance in solution, and (6) to be without organic contamination.

Except the purest rain-water, an absolutely colourless water does not exist in nature. Potable waters all have a certain degree of colour when viewed *en masse*. The tints of colour usually vary from bluish to yellowish; the former in chalky waters, the latter in peaty waters from upland lakes. Upon rare occasions a greenish hue is observed in water taken from rivers, reservoirs, or lakes infested with pond-weed, which is due to the dissolved green-colouring matter of the plants. Water of a reddish tint betokens iron. Good water is palatable, does not possess any distinct taste. But palatability may be found in an impure water. This characteristic depends upon the presence of the atmospheric gases in the water; when water is boiled, and these gases are driven off, the water becomes insipid to the average palate. Actual taste in a water can only be imparted either by (1) contamination, or (2) by an unusual amount of mineral substances present—as in mineral waters. We have examined a water which tasted of petroleum, due to accidental contamination of the reservoir. The substance which, in smallest amount, imparts taste to a water, is iron, the amount being one-fifth grain per gallon. Lime salts do not taste a water till they exist in amounts from 10 to 25 grains per gallon. Before common salt can be tasted, it must be present to the extent of 75 grains per gallon. Certain water plants impart a bitter taste.

Good water has no smell. Certain mineral waters have a disagreeable odour owing to free sulphur gases. Water, however, may be accidentally contaminated by fæcal matters, or by coal gas, which are revealed by the odour. We once examined a water which smelled of coal gas. The best way to detect an odour in a water is to heat it. No water is usable which has a perceptible odour. A water which has no colour, taste, or smell, is usually also bright, sparkling, and clear. But this last feature is often found in contaminated waters, hence not much reliance must be placed on this single characteristic, as gases of decomposition may simulate the appearance of atmospheric gases. The presence of lime and magnesia may be predicated by the hardness or softness. The "usability" of a water does not so much depend upon the amount of the mineral substances present—although to some extent it is a determining factor—as upon their nature. Certain of them are considered as indicative of animal contamination; and the amounts found which are sufficient to condemn a water are, comparatively speaking, infinitesimal. They act as the danger signal. Such are the chlorides, the nitrates, and the salts of ammonia. They are not hurtful *per se*, but they often point to organic impurity, to sewage contamination, and to likely accompanying hurtful elements. All waters which contain organic matter are suspicious until it be determined whether the source of it be animal or vegetable; the latter, except when considerable, being comparatively innocuous. Animal organic impurity is dangerous.

All the foregoing considerations point to the need for continuous and careful examination of water supplies, both chemical and biological; and also, for prudent deliberation in the introduction of a new supply, both in respect of (1) purity, and (2) adequacy and continuity of supply.

Effects of Impure Water on Health.—Impurities in water apt to affect health are divisible into two classes, viz., (1) those which have their source in filth or sewage contamination, and (2) those due to the nature and amount of mineral constituents. The second class is of lesser vital importance than the first. From the former may arise dysentery, cholera, enteric fever, and diarrhœal diseases. Not many years ago about 20,000 inhabitants of Hull were more or less affected by reason of ditch water finding its way into the supply reservoir, and about 1,000 cases of enteric fever occurred in Kidderminster from the accidental leakage of a sewer into the public water-supply. From a newly-manured field, the surface drainage entering the water-supply, 400 cases of the same disease resulted; and a like outbreak, attributed to Glasgow manure, although not so extensive, occurred near Irvine, in the West of Scotland. Water contaminated with the specific microbe of enteric fever and mixed with milk has been the provocative cause of several epidemics of this disease in Glasgow and elsewhere. In fact, the farmyard well has done more to propagate this disease in cities than any other single cause, if not indeed than all causes put together. Hitherto too little attention has been paid to the sanitation of farmyards, either in respect of milk storage or milk pollution. Cisterns for fresh water are apt, through careless or ignorant plumbing, to become contaminated. We have seen cases to which illness from this cause was clearly traceable, and at Caius College, Cambridge, where an outbreak of enteric fever among the students occurred, the same cause obtained. From the foregoing, therefore, it will be observed that a water-supply is apt to become polluted either (1) at the source of supply; (2) during conveyance; or (3) at the point of distribution. To avoid contamination it is necessary to select a "gathering ground" for the water supply removed as far as possible from the haunts of men, and to safeguard it against sources of pollution.

Cholera is most usually a water-borne disease. The recent outbreaks at Hamburg and elsewhere demonstrate the relation between this disease and a water contaminated by the specific microbe. A pure water supply, therefore, is the first and best line of defence against cholera.

Certain parasitic diseases which affect man are also traceable to water which contains the ova of certain parasites. Intestinal worms have, not infrequently, this origin, and are found both in man and in the lower animals. During the tunnelling of Mount Gothard many of the workmen were seized with intestinal hæmorrhages, which, after investigation, were found to have been caused by microscopic leech-like parasites in the drinking-water.

The mineral constituents of water, when abundant, are apt to create, not only discomfort, but actual disease. It is sometimes difficult to believe that alterations in diet, or physical surroundings, affect health, and it is equally so to think that the mere change of water-supply may be the cause of actual illness. Persons who, for nine or ten months of the year, use for drinking purposes, and in the preparation of food, soft water, are very liable at first to gastro-intestinal disturbance when they remove for a summer holiday to a district where the water is hard. This is a common experience. Constipation, alternating with diarrhœa, or continuous diarrhœa, are common effects, until acclimatisation is effected. Certain diseases have been for ages attributed to the constant use of hard water. "Derbyshire neck," or goître, is one of these, and cretinism—chiefly found in Switzerland—is another; they are not infrequently found associated. By some observers they are said to be due to the sulphates of lime and magnesia, and by others to certain metallic sulphides, notably that of iron. In Switzerland, the Himalayas, Cochin China, and the Pyrenees, cretinism, which consists of a certain rickety condition of the bones of the body, associated with mental imbecility, is by no means uncommon. A French Commission appointed to inquire into this subject obtained some striking evidence on this subject, as, for instance, the following: In 1848, in a town called Bozel, of a population of 1,472 persons, 900 had gôitre and 109 were cretins, while the population of a village, St. Bon, situated about 1,100 yards higher than Bozel, was absolutely free from both diseases. Each place had a different water supply, that of the former a "hard" water containing magnesium and lime sulphate, that of the latter a "soft" water. A pipe was laid from St. Bon to Bozel conveying this soft supply. Sixteen years after—1864—Bozel only contained 39 gôitrous persons and 58 cretins. While the connection between hard water and gôitre is so close, it has not yet been absolutely determined that lime and magnesium salts are alone the causative agents. But they have this connection, although their physiological operation in the body is still obscure. Vegetable matter—principally from peat—when present in amount may give rise to gastro-intestinal catarrh, but more serious mischief is not likely to follow.

### Trained Nurses' Clinic.

### IX.—OPERATIONS.

The danger that sympathetic care of the individual may be merged in professional interest in "the case" is seldom so imminent as when there is an operation in prospect. When future health, and perhaps even life itself, depend on the results of the surgeon's operation, a nurse may well give the most conscientious attention to every detail of her duty; and most thoroughly trained women can be trusted to attach due importance to the preparations which lie within their special province, otherwise they would soon cease to be acceptable surgical nurses.

Modern progress in surgery naturally necessitates the possession of a great deal of practical and technical knowledge by those who have even the most subordinate connection with the performance of operations, and all will acknowledge the need for the thorough training of every faculty of the woman who desires to excel in this branch of a nurse's calling. It is, however, well to bear in mind that there is some risk of such preparation becoming scientific rather than humane. The ambition to understand the details of the surgeon's work may seem laudable, but it must be borne in mind that any such comprehension on the part of a nurse can only be superficial after all.

Observations based on proceedings imperfectly seen are naturally inaccurate, and in the case of a nurse, whose duty, during the operation, consists exclusively in serving others, they must be often quite unreliable. The operator acts on a knowledge acquired through long years of hard work and of study. He has precedents for most of his actions; he has had opportunities and education such as fall to the lot of none but experienced and duly qualified medical men. All these preliminaries are somewhat overlooked by the nurse who aspires with doubtful success to be considered the surgeon's assistant.

It is but a natural outcome of modern training that the surgeon finds himself supplied with an intelligent and valuable helper. In place of the rough-handed obsolete scrubber of old days, a new type of nurse has been evolved. The old-time woman could see the beauty of scrupulous cleanliness for deal flooring, but of what is now called "surgical cleanliness" she had practically no knowledge. The modern surgical nurse is absolved from the labour of scrubbing boards, but she is expected to carry out such an elaborate purification of many other things as would be inconceivable to her sisters of past days. Yet with all this acquired knowledge the nurse is still the *nurse*; she has no claim whatever to the place of medical student, much less that of assistant. A clever nurse takes a natural and justifiable pride in the dependence placed on her by the surgeon, but her ability seldom extends beyond her own limited sphere, and it not unfrequently fails to show her the contempt into which she brings the calling of nurse if she chance to exhibit even temporary forgetfulness of the subordinate position which she herself must hold.

Although the fact is the same in both medical and surgical nursing, it is in the latter that there appears most fear of the occurrence of the danger pointed out in the commencement of this article.

The smart, quick nurse, who can render herself almost indispensable in the operating-room, and hence earns golden opinions from the accomplished surgeon (who has no further acquaintance with her), may be most unacceptable to the patient. The latter is ignorant of all that took place whilst he was "on the table," but he is a very good judge of the way in which he is afterwards regarded by his attendant.

The most unobservant persons are keenly alive to the absence or presence of sympathy in those who look after them in illness; in fact, they are swift to recognize the distinction between their treatment as cases or individuals.

Any nurse who allows a patient to feel that her interest in her work ceases, when the excitement of an operation is succeeded by uneventful convalescence, must injure the whole nursing body. She obtains a reputation for hardness, which may or may not be deserved, and she creates in her patient's mind and that of his friends a conviction that a love of operations is the leading characteristic of nurses, and that probably it exists to the exclusion of humane and womanly consideration for the individual.

### Where to Go.

Bovril and Company.—Lord Playfair and the directors of Bovril are inviting nurses to inspect their factory and observe their process of preparing Bovril, on Tuesday, July 28th, at 63, Bath Street, City Road, from three to six o'clock.

Royal British Nurses' Association. — The Secretary requests us to announce that the annual meeting of members of the Royal British Nurses' Association will be held in the Great Hall of St. Bartholomew's Hospital, E.C., on Wednesday, July 22nd, 1896, at half-past eleven a.m. Notice of the following resolution has been given by Miss Margaret Breay: "That this meeting of the Corporation expresses its strong disapproval of the methods of management pursued by the present Executive Committee, especially concerning the manner in which the pledges given by the Association have been broken; in which the expenditure of the Association has been allowed so greatly to exceed its reliable income; in which the provisions of the charter and bye-laws have been violated; and in which a member of the Association has been compelled to appeal to the Court of Chancery for protection against the Executive Committee."

### Wants and Workers.

[The attention of correspondents is directed to the fact that "Helps in Sickness and to Health" (Scientific Press, 428, Strand) will enable them promptly to find the most suitable accommodation for difficult or special cases.]

Home wanted for girl of 15, partially imbecile (quite harmless) and crippled from hip disease. No payment could be made. The girl can do light house work, and is capable of being taught. Address Nursing Sister, care of the Editor, 428, Strand, W.C.

### Our American Letter.

THE New York City Training School for Nurses, Blackwell's Island, celebrated its twenty-first annual "commencement" on June 5th. From the river the corner of the island where the nurses' home stands looked most attractive, illuminated with Chinese lanterns. After the diplomas had been presented to the graduate nurses, and Miss Darche had read her annual report, a reception was held, attended by many guests.

On June 15th, Miss M. Isabelle Merritt, superintendent of the Training School for Nurses of the Brooklyn Hospital, presided at a meeting held at the Hoagland Laboratory to perfect the organisation of the Associate Alumnæ of the Brooklyn Training Schools. Miss Merritt explained that the constitution of the Association had been most carefully compiled, with a view to making it as fair on all sides as possible. The intentions of the association are to foster all movements to improve the nursing interests, to form a central registry, and to establish a proper business connection with the medical profession and people of Brooklyn. About fifty nurses were present at the meeting, and by them the constitution of the association was voted upon and finally accepted without change. Miss Merritt was unanimously elected President, and Miss Ida Sutliffe, of the Long Island Training School, Miss Marcella Doyle, St. Mary's Hospital, and Miss Black, Memorial Hospital, were elected vice-presidents.

Thirty-three graduate nurses received diplomas at the Johns Hopkins Hospital Training School lately. Addresses were given by Dr. John Musser, of Philadelphia, and Dr. Henry M. Hurd, the latter presenting the nurses with their diplomas. Miss Nutting read the annual report of the school, and a reception in the home followed. Miss Nutting lately read a very interesting paper on nursing on the occasion of the graduating exercises at the Garfield Hospital.

Improvements are being gradually effected throughout the United States in the general accommodation provided for nurses. The authorities of the various training schools have become impressed with the importance of providing proper and separate buildings where their nurses may retire for sleep and recreation, enabling them to leave hospital atmosphere entirely behind them and so return refreshed and invigorated to their work. Several hospitals now possess charming nurses' homes. The Anna Jaques Hospital at Newburyport, Massachusetts, has now a delightful building for its nurses, erected with a sum of money left by the late Mrs. Lucy Moseley Muzzey for some charitable purpose. The Rochester City Hospital is hoping to make the much-needed addition of a home to its buildings without loss of time. The managers of the Friends' Asylum for the Insane, Philadelphia, and of the Presbyterian Hospital, New York City, are issuing appeals for the same purpose, which it is to be hoped will meet with a prompt response from their friends.

St. Louis is suffering much at the present time in the loss of the City Hospital by the late terrible cyclone. The old building, erected fifty-three years ago, is a wreck, and in the small portion left standing there is not one useable room. Various plans are on foot for the erection of a new hospital as quickly as possible, one suggestion being to tear down what remains of the old one and begin erecting the series of one-storey pavilions (the proposed form of the new building) at once, one at a time, as fast as the ground is cleared. Lafayette Park is talked of as an alternate site. In the meantime the old Good Shepherd Convent, kindly lent as a temporary hospital in the stress and bustle after the storm, is found be inadequate to the demands upon its space, and Dr. Sutter has suggested the use of the old buildings of the Missouri Blind Asylum for temporary use.

A wonderful new ambulance will complete the equipment of the new isolation hospital now being built at Chicago; it will be fitted with all the latest devices for comfort and convenience, electric light, swinging couches, and other arrangements for making easy the removal of the sick. Two Chicago citizens, Mr. H. Kohlsaat and Mr. Armour, have just presented generous gifts to the Provident Hospital Association of that town. Mr. Kohlsaat has given a site at the north-west corner of Thirty-sixth and Dearborn Streets, and Mr. Armour has undertaken to defray the cost of erection thereon of new hospital buildings for the benefit of the sick poor among the coloured population of Chicago.

### The Colonial Nursing Association.

THROUGH the untiring exertions of Mrs. Francis Piggott, a scheme has been set on foot which aims at providing much-needed skilled nursing for our colonies. Ernestly impressed with the requirements of English communities in our Crown colonies, Mrs. Piggott has worked out a scheme for which she has succeeded in enlisting warm and influential support, and only needs the necessary funds to set the association she has formed actively at work. Lord Loch has consented to act as president, and Mrs. Joseph Chamberlain, the Viscountess Knutsford, Lady Lucy Hicks-Beach, Lord Stanmore, Mr. W. Rathbone, and many other influential ladies and gentlemen are amongst the vice-presidents. Miss Rosalind Paget and Mrs. Francis Piggott are on the committee of management, the latter acting as hon. secretary also. The objects of the association are to raise funds from subscriptions and donations to assist colonial communities to secure skilled nurses when they are unable to pay preliminary expenses, and in some cases wholly to support them. It is hoped that eventually the scheme will become self-supporting, and that pecuniary aid will only be required from the association in the first or occasional instances. It is intended to supply a nurse or nurses to representative local committees, who will be charged with securing the necessary funds, and bearing all expenses as soon as possible. Nurses will be required to enter into an agreement for not less than two years, salary, maintenance, and travelling expenses being provided. There can be no question that nurses are much needed in the colonies, the representations of medical officers testifying to the fact that in many cases loss of life could be prevented if skilled nursing were procurable. It rests with an association to do the work necessary, as individual nurses are not inclined to, and indeed they are frequently warned against taking up private nursing abroad. Working for the association the risk is removed, and the conditions are rendered favourable to the nurse, and afford security to the public. The address of the association is Colonial Nursing Association, Imperial Institute, S.W.

On Wednesday, the 15th inst., a drawing-room meeting, at which Lord Loch presided, was held at the residence of Mr. Cuthbert Quilter in support of the scheme.

### St. Olave's Union Infirmary.

WE regret to find that a printer's error has crept into the account respecting this infirmary and its matron, which appeared in last week's *Mirror* on p. cxxvii. In the letter from the Local Government Board to the Board of Guardians, dated June 30th, on the eighteenth line, the word "Board" has been substituted for "Matron." The sentence should have run: "The Guardians have evinced a want of due consideration for the matron's position." This meaning must have been obvious to all careful readers, but in case any doubt may have arisen we reiterate the remark of the Local Government Board.

### Dress and Uniforms.

By a Matron and Superintendent of Nurses.

### KING'S COLLEGE HOSPITAL.

King's College Hospital has long been famous, and justly so, for the high standard maintained by its nursing staff. It is a fact which is patent to anyone who enters this quaint, may serve as a hint to those nur.es who adopt either unconsciously or purposely those free and easy gestures, such as arms held behind the back, akimbo, or in the pocket, which are so unbecoming in a sick ward. A dress of dark green

Probationer. Sister. Staff Nurse.

time-honoured institution. The neatness with which each one wears her uniform, the refined manner and graceful deportment, are all evidence of the careful attention paid to the little nameless trifles which go to make the sum total of what is comprehended in a perfect nurse. In the group before us these characteristics are admirably portrayed. The attitudes, while strictly professional, are easy and natural enough, to avoid any suggestion of restraint, and merino, of that peculiar shade known as raphal green, is worn by the sisters of this hospital. The skirt is made full and just off the ground, and is connected with the bodice by a band at the waist. The sleeves and back are relieved by plain bands of white linen. A white apron of ample width covers the dress, and is provided with a square bib fastening into the bodice at each corner. The cap, which is of the "Sister Dora" shape, is made of bishop's lawn, and ties with narrow strings in a neat little bow under the chin. Blue and white striped galatea is the material of which the staff nurses and probationers' dresses are made. The skirt and bodice is quite plain, and the sleeves coat shaped. White linen aprons are worn with bib and straps, which cross at the back and fasten into the band at the waist. The staff nurse's cap is similar in style to those worn by the Normandy peasants, and makes a becoming finish to an attractive costume. The probationer's cap is of spotted net edged with a deep gophered frill of the same, drawn into position by a thread run on the reverse side. This is what is usually known as the Nightingale cap, which is as simple as it is becoming. Our readers will agree with us that the group we have just described is an exceptionally pleasing and interesting one.

### Everybody's Opinion.

### QUEEN'S NURSES AT WINDSOR.

THE MATRON OF THE MANCHESTER AND SALFORD SICK POOR AND PRIVATE NURSING INSTITUTION writes: May I be allowed to correct a slight error in your most interesting account of the "reception of 'Queen's Nurses' by Her Majesty at Windsor"—a day, indeed, long to be remembered by all of us? In speaking of the uniform, it is stated that "Manchester nurses wore green cloaks and bonnets, instead of the regulation blue." There are five affiliated homes in Manchester, all under the Manchester and Salford Sick Poor and Private Nursing Institution. It is only in one of these where green is worn; in all the others the recognised "Queen's" uniform is strictly adopted. I am anxious for this to be distinctly understood, as we are proud of the "regulation blue."

### TRAINING AS CHILDREN'S NURSES.

THE MATRON of Moseley Hall Convalescent Hospital for Children, near Birmingham, writes: Noticing in THE HOSPITAL of last week an account of the Norland Institute, may I ask you to insert a paragraph about this institution which has a somewhat similar end in view. We train young ladies as nurses in gentlemen's families, or prepare them for nursing in large hospitals, where they often break down in their first year for want of some preliminary training. This hospital has sixty-six beds which are occupied by patients from the hospitals, dispensaries, and medical charities of the city. It stands in charming grounds, and is managed by a sister and staff nurses who have had special training in children's hospitals. The probationers have the usual training under more healthy surroundings than can be obtained in large hospitals, and they have, combined with that, a feeling of home and of home comforts. The rudimentary nursing knowledge makes a nurse valuable in private families, and should be invaluable to those who are going on to busy hospitals, where the head nurses can be saved from the trouble of giving elementary training to her probationers.

### MIDWIVES AND MIDWIFERY NURSES.

"MIDWIFE" writes: Some time back a leading article appeared in your paper with regard to nurses being, instead of helps, rivals to doctors. I had then no time to take up the question and answer it. But perhaps it would be as well to give a hint to small general practitioners and country doctors. Let them treat us with the same courtesy and consideration as the "big men" do, and then we would be more likely to be loyal to them and show them, outwardly at least, deference and respect as to a superior, though we may not feel it in our inner hearts. Unfortunately, a capable nurse does know the condition of a patient, and whether the prognosis is favourable or otherwise, quite as well as the doctor, and by living in the house wins the confidence of the patient and friends. It may be disloyal of a nurse to express an opinion, but she would need to be a stoic not to betray herself; besides, she is often asked her opinion independently of the doctor. Should she refuse to give it? I have known two nurses dismissed on that account. What reward did they get from the doctors? They (the doctors) were told by the patients, in the first instance, that the nurse was sent away "because she did not know or understand my case"; in the second, "That nurse was a nasty disagreeable stick—a hired machine." What has this to do with my heading, you will say? This, that all this fuss about the legislation and registration Bill is simply and purely jealousy and rivalry on the part of doctors—not the "big men," again; they can afford to give us a helping hand, because we do not interfere with their practice; they only know us as nurses, not as midwives, and we are not a thorn in their flesh as we are to the struggling practitioner, who through us loses fees. But should we cross their paths and do them a little harm, we do a great deal of good on the other hand, for we are gradually doing away with the quack midwife and Gamp nurse, and thus, surely, the good exceeds the evil. Such as we are, we are better than the untrained woman. As to calling us "midwifery nurses," that term would be more correct with the "L.O.S.," who nurse private cases under a doctor, but you cannot call a midwife who delivers without medical aid a "midwifery nurse." Looking upon things from a broad, just point of view, I think that as things stand now they are as they ought to be, and that we ought to be called midwives. We deliver all normal cases, and even if we do have to send for medical advice and help in all cases of difficulty, why our position towards the medical man is a clear one. We are midwives and they are doctors! A midwife is inferior to a doctor, therefore, when she works under him in private houses, and when she sends for him in cases of difficulty in the district, both she and her patients understand clearly that in his presence she stands second—he takes the responsibility and she his orders. Why, therefore, this newfangled word, "midwifery-nurse," and why all this delay in deciding our future position? We exist; they cannot do away with us; let them make the best of us such as we are. A midwife who is a trained nurse naturally is a more highly qualified one than a simple midwife, but are all medical men equally qualified? Call us "midwives," and the public will understand what you mean—it sounds familiar, and "midwifery nurse" strange and newfangled, and would be incorrect in all but private nursing cases.

### Royal British Nurses' Association.

THE quarterly meeting of the General Council of the Royal British Nurses' Association was held on Friday, July 10th, 1896. H.R.H. Princess Christian of Schleswig-Holstein, president of the association, took the chair, and some thirty or forty members were present. The Medical Hon. Secretary presented the report of the Executive Committee, which included the authorisation of the formation of a colonial branch of the association in New Zealand, and a proposed course of practical demonstrations in invalid cookery at the offices of the association during the winter months. Dr. Outterson Wood raised a question as to the advisability of the Royal British Nurses' Association admitting as members thoroughly trained mental nurses, as such, *i.e.*, as a distinct class, and after some discussion the consideration of this important matter was referred to a special sub-committee, which should consider the subject in all its details, and report the result of their deliberations to the council at its next meeting.

### Minor Appointments.

TAUNTON SANITARY HOSPITAL.—Miss Marian Bardwell has been appointed Nurse-matron of the above hospital. She was trained at the Halifax Fever Hospital, and held an appointment at the Sanitary Hospital, Bournemouth, subsequently.

ST. KATHERINE'S COTTAGE HOSPITAL, CLUN.—Mrs. J. Hymus has been appointed Nurse-matron of the above institution. She was trained at Addenbrooke's Hospital, and subsequently held the post of matron at Frome Cottage Hospital for five years, at Reigate Cottage Hospital for one year, Brentwood for a year and a-half, and Abergavenny for three years. Her experience, therefore, in the management of cottage hospitals is exceptional.

### The Book World for Women and Nurses.

[We invite Correspondence, Criticism, Enquiries, and Notes on Books likely to interest Women and Nurses. Address, Editor, THE HOSPITAL (Nurses' Book World), 428, Strand, W.C.]

### MAGAZINES OF THE MONTH.

THE WINDSOR MAGAZINE.

THIS month's is the best number of the above magazine which has yet appeared, a magazine which has fully maintained the success which it gave promise of in its earlier days. "Some 'Varsity Blues" and "Muscular Christianity" form, perhaps, the two most attractive features in its present issue. The writer of the latter article points out with a circumstantial elaboration the close connection between "theology and oarsmanship," and, as one example (among many), he mentions "the first occasion when a picked crew of Oxford tried conclusions with the oarsmen of Cambridge, when four of the Dark Blues who sat in the boat that memorable 10th July, 1829, were destined presently to become a Bishop, two Deans and a Canon."

CASSELL'S FAMILY MAGAZINE.

We never take up this journal without some educational advantage accruing therefrom. The July number proves no exception to the rule; it is full of readable matter from cover to cover.

PEARSON'S.

Very charmingly got up is the July Pearson, and a pleasant, bright *compagne de voyage* it will prove for those who are holiday-making this month; it has a pretty attractive cover printed in colours, and the illustrations throughout the pages are of exceptional excellence. There are several short stories and other articles which raise the magazine above any ordinary mediocrity.

CORNHILL MAGAZINE.

WITH the July number of the CORNHILL MAGAZINE a new series begins under new editorship, and, whilst the original price is reverted to, the magazine is increased in size. Mr. Henry Seton Merriman will supply the next serial story after the conclusion of Mr. W. E. Norris', which is now running on. In the present issue there is an interesting article on "The First Number of the 'Cornhill,'" by Mrs. Richmond Ritchie, in which several unpublished letters of the writer's father, Thackeray, appear for the first time. "The CORNHILL was a fine performance from its first commencement," as Mrs. Ritchie remarks, "deserving well of this country, the names of the contributors being a sort of history of the doing and thinking of the action of philosophy from the year 1860." Other items in this number of the magazine are an "Anniversary Study of Burke," "Memoirs of a Soudanese Soldier," "A Lottery Duel," "Black Ghosts," &c. We congratulate the editor on the general improvement of the new series.

THE SUNDAY AT HOME.

An illuminated text frontispiece opens the pages of this magazine. Mr. Leslie Keith contributes a short story, "The Peacemaker," which is complete in this number. "Winchester Yesterday and To-day" is a charming and instructive paper on an unusually interesting old English city, the illustrations of which are very good. Two papers on "The Handwriting of Famous Divines" have an interest for those who pursue the now fashionable study of caligraphy. Bishop Jeremy Taylor's is dealt with in the present issue, and a full-page specimen is given of one of his letters.

---

The GIRLS' OWN PAPER has a full and varied programme of contents for July. Fiction, poetry, varieties, recipes, cookery, dressmaking being amongst the subjects dealt with. Of topical interest are the articles on "How I Learned to Bicycle," by Constance Hastings, and "Life on a Transvaal Salt Farm."

In the same way that "The Girls' Own Paper" is a wonderful sixpennyworth, so also is THE BOYS' OWN PAPER, from the same office. It is equally well written, illustrated, and edited with a view to its requirements for young people, and the subjects are thoroughly up to date. "Skeletonic Photography," by R. H. R. Bennett, is described by pen and pencil in a comprehensive manner, and the same issue contains useful hints on "Home Carpentry." There is a complete short story, and a fine coloured frontispiece, "When Jack's at Sea."

### Presentations.

ON resigning her position as matron at the Royal Sea Bathing Infirmary, Margate, Miss Carew Oxley received from the nursing staff a set of silver spoons. The committee gave her on the same occasion an illuminated address, which is engrossed on vellum, and runs as follows: "The Court of Directors, on the suggestion of the Executive Committee, take this opportunity of recording their regret at Miss Oxley's resignation, and of thanking her for her valuable services since she has been the matron of the institution, in cordially co-operating with the Court of Directors in carrying out the many necessary and vital reforms required for the well-being of the institution, and they beg to offer her their congratulations and hearty good wishes on the occasion of her approaching marriage."

### Notes and Queries.

The contents of the Editor's Letter-box have now reached such unwieldy proportions that it has become necessary to establish a hard and fast rule regarding Answers to Correspondents. In future, all questions requiring replies will continue to be answered in this column without any fee. If an answer is required by letter, a fee of half-a-crown must be enclosed with the note containing the enquiry. We are always pleased to help our numerous correspondents to the fullest extent, and we can trust them to sympathise in the overwhelming amount of writing which makes the new rules a necessity. Every communication must be accompanied by the writer's name and address, otherwise it will receive no attention.

### Queries.

(106) *The Visiting Nurse.*—Some little time ago I saw some correspondence in your columns on this subject. Would you give me the name and address of the lady who, I understood, has been working such a scheme with success?—*F. H. L.*

(107) *Wardmaids.*—How can I get into a hospital as housemaid or wardmaid? I have been housemaid in private families, but am very anxious to get into a hospital.—*E. K.*

(108) *Queen's Nurses.*—Where can I get information about the Queen's Jubilee Institute for Nurses?—*Nurse Fanny.*

(109) *Nurse Helen.*—Can pupils be trained in midwifery for the L.O.S. examination in the provinces, and how can I obtain particulars of this examination?—*L.O.S.*

(110) *Training.*—Where can a young girl of the artisan class be trained as a nurse that she may nurse her own people?—*E. E.*

(111) *District Nurse.*—Will you tell me if any district nurse in England is working for less than 14s. a week, paying her full board and lodging and washing, &c., all the year round, taking no meals in the patients' houses? I find the above does not cover my expenses, but am told I am getting better pay than some nurses.—*E. H.*

(112) *Newcastle.*—Can you tell me where a young man, who is imbecile and a deaf mute, can be received? Maximum payment 5s. weekly.—*Deaf Mute.*

### Answers.

(106) *The Visiting Nurse (F. H. L.).*—Write to Miss C. J. Wood, Nurses' Hostel, 27, Percy Street, W.C.

(107) *Wardmaids (E. K.).*—Look at the advertisements each week in the "Nursing Supplement" of THE HOSPITAL, or apply personally at any of the hospitals. If you are strong and suitable in other ways you will not find any difficulty in getting such employment.

(108) *Queen's Nurses (Nurse Fanny).*—You will have seen the address of the Jubilee Institute in THE HOSPITAL for last week. Write to the Secretary, Queen Victoria's Jubilee Institute for Nurses, St. Katharine's Hospital, Regent's Park, N.W.

(109) *Nurse Helen (L.O.S.)*—Yes. Get "How to Become a Nurse," Scientific Press, 428, Strand, W.C., if you want a list and particulars, and for the last you can write to the Secretary, L.O.S., 20, Hanover Square, W.

(110) *Training (E. E.)*—Numbers of provincial general and cottage hospitals receive probationers for small fees (or without). You do not mention period of training required. Burdett's "Hospitals and Charities" and "How to Become a Nurse" give lists.

(111) *District Nurse (E. H.)*—We cannot say whether any nurse receives less than 14s. a week, but that sum is too small. The East London Society give their nurses 15s. to 18s. a week, and provides uniform and furnished lodgings. The Metropolitan and National Association gives £35 to £50, and all found; and we are of opinion that no district nurse should accept a salary of less than £50 if she has to find everythidg, seeing that she has no time to attend to small comforts and economies for herself.

(112) *Newcastle (Deaf Mute).*—The case is most difficult. You will find a list of establishments and advice in "Helps in Sickness and to Health" (The Scientific Press, 428, Strand), and by making systematic application to the various establishments we hope you will find what you need, but we fear you will have a difficult task before you.

## Figures and Tables

**Figure f1:**